# Construction of CII-Specific CAR-T to Explore the Cytokine Cascades Between Cartilage-Reactive T Cells and Chondrocytes

**DOI:** 10.3389/fimmu.2020.568741

**Published:** 2020-12-04

**Authors:** Xiaolong Liu, Jun Zhao, Ce Shi, Zhiyu Liu, Hongtao Shen, Junlong Dang, Yang Li, Dongguang Yang, Jia Wei, Liqing Kang, Jin Zhou, Fenglin Cao, Song Guo Zheng, Zhenkun Wang

**Affiliations:** ^1^ Central Laboratory, First Affiliated Hospital, Harbin Medical University, Harbin, China; ^2^ College of Life Science, Northeast Agricultural University, Harbin, China; ^3^ Department of Clinical Immunology, Third Hospital of Sun Yat-sen University, Guangzhou, China; ^4^ Department of Orthopedic Surgery, First Affiliated Hospital, Harbin Medical University, Harbin, China; ^5^ Division of Rheumatology, Department of Medicine, Penn State College of Medicine, Hershey, PA, United States; ^6^ Institute of Biomedical Engineering and Technology, Shanghai Engineering Research Center of Molecular Therapeutics and New Drug Development, School of Chemistry and Molecular Engineering, East China Normal University, Shanghai, China; ^7^ Department of Hematology, First Affiliated Hospital, Harbin Medical University, Harbin, China; ^8^ Department of Internal Medicine, Ohio State University College of Medicine, Columbus, OH, United States

**Keywords:** cytokine cascade, type II collagen, chimeric antigen receptor T cell, inflammatory arthritis, cartilage

## Abstract

Cytokine cascades exist in many autoimmune disorders which amplify and sustain the autoimmune process and lead to chronic inflammatory injury to the host tissues. Increasing evidence indicates that chondrocytes can interact with T cells, which may be a crucial event in inflammatory arthritis. To address the reciprocal influences of cartilage-reactive T cells and chondrocytes, we constructed cartilage-reactive T cells by developing a type II collagen-specific chimeric antigen receptor (CII-CAR). An *in vitro* co-culture model of CII-CAR-T cells and fresh cartilage was developed, in which CII-CAR-T displayed specific proliferative capacity and cytokine release against fresh cartilage samples, and chondrocytes could respond to CII-CAR-T cells by secreting IL-6. The proposed model will help us to explore the possible cytokine cascades between cartilage-reactive T cells and cartilage.

## Introduction

Cytokines are important mediators of immunity and major drivers of autoimmunity. Once the autoimmune process has been triggered, the cytokine cascades occur and play an important role in the pathogenesis (1). Inflammatory arthritis, such as rheumatoid arthritis (RA) is one of the main diseases that cause the loss of labor and disability in the population (2, 3). The cytokine network in RA is complex; pro-inflammatory cytokines, including tumor necrosis factor (TNF)-α, interleukin (IL)-6 and the mediators produced through downstream pathways constitute the milieu driving neoangiogenesis. The neoangiogenesis can lead to the infiltration of a large number of inflammatory cells in the joint (*eg*, T cells, B cells, macrophages, and neutrophils), which will further cause cartilage destruction and bone erosion, and eventually lead to joint deformities and dysfunction (4–6). Especially T cell-mediated inflammation is closely related to the occurrence and development of RA (7–10).

Under pathological conditions, T cells behave as a hub, in which B cells, dendritic cells (DCs), and tissue-resident cells can interact with T cells to intensify the process of RA (11–14). Recent studies have shown that chondrocytes play an important role in amplifying inflammatory responses during RA development (15). Exploration of the amplifying inflammatory responses produced by cartilage-reactive T cells and chondrocytes might help reveal the effective clinical interventions and treatment targets.

Both *in vivo* and *in vitro* models can be used for studying disease mechanisms and preclinical testing, and play an appropriate role in different situations. A simple but appropriate *in vitro* model mediated by T cells and cartilage that enables investigation into the cytokine cascades might be used as a suitable and rapid tool to develop strategies for inflammatory arthritis therapeutic application.

In this study, chimeric antigen receptor (CAR) was used to construct easily obtainable cartilage-reactive T cells. Chimeric antigen receptor-T cell (CAR-T) therapy is a novel immunotherapeutic approach for treating cancer, with exciting initial successes targeting hematologic malignancies (16, 17). The most prominent and serious toxicity of CAR-T cell therapy is cytokine release syndrome (CRS), a systemic inflammatory response caused by cytokines such as IL-2, IL-6, TNF-α, IFN-*γ*, *etc.*, and these cytokines also play a role in the occurrence and development of inflammatory arthritis and other autoimmune diseases (18–20). Swelling and pain in the large joints of the limbs which similar to RA was reported in patients with CAR-T therapy (21). Type II collagen (CII) is the main structural protein of articular cartilage, accounting for about 85–90% of cartilage collagen (22). Although CII is sequestered from the immune system under normal physiological conditions, it can be exposed as a target for autoimmune specific attack during the pathological process of RA disease progression (23). To facilitate direct reflection of T cell-targeted cartilage interactions, we constructed an anti-CII single-chain antibody fragments (scFv)-CD137-CD3ζ second-generation CAR vector (CII-CAR) and obtained CII-CAR-T cells by lentivirus infection of CD3^+^ T cells in this study. These CII-CAR-T cells can target cartilage to produce proinflammatory cytokines such as IL-2, TNF-α and IFN-*γ*, *etc*. These cytokines are also widely present in the joint fluid of patients with RA (24, 25). Thus, we have established a rapid model of T cell-mediated inflammation *in vitro*, which provides a suitable experimental tool for studying the cytokine cascades caused by the interaction between cartilage-reactive T cells and chondrocytes.

## Materials and Methods

### Cell Lines

Cell lines C28/I2 and 293T were generously provided by Dr Qiao (School of Life Science and Technology, Harbin Institute of Technology, Harbin, China), and cultured in Dulbecco’s Modified Eagle’s Medium supplemented with 10% fetal bovine serum (FBS, Gibco) at 37 °C incubator in a 5% CO_2_ atmosphere.

### Human Blood and Articular Cartilage Samples

Human blood samples were obtained from healthy donors ranging from 18 to 65 years of age. Peripheral blood mononuclear cells (PBMCs) were isolated using Ficoll-Hypaque (GE Healthcare Biosciences) by gradient centrifugation. Cells were then frozen in FBS containing 10% dimethyl sulfoxide (Sigma-Aldrich) and kept at −80°C. Cartilage was obtained from six eligible male subjects undergoing total knee arthroplasty for osteoarthritis (age range 56–66 years, median age 57 years), and cartilage were obtained from normal-appearing areas of discarded tissues according to the method of Zhou et al (26, 27). They were cut to circular pieces with a 3.0 mm tissue punch and placed at the bottom of 96-well plates for subsequent experiments. Freeze–thawed cartilage (FT-cartilage) samples were subjected to three rounds of freeze–thaw cycles at liquid nitrogen and 37°C water bath to kill the chondrocytes (28, 29). The study was conducted subject to the approval of the Institutional Review Board of Harbin Medical University and in accordance with the Declaration of Helsinki. Discarded articular cartilage after surgery and peripheral blood samples were taken with written informed consent of patients and donors.

### Collagen II Immunofluorescence Staining

C28/I2 was cultured in 24-well plates; after fixing in 4% paraformaldehyde for 10 min, cells were permeabilized with 0.5% Triton X-100 at room temperature for 20 min and sealed with bovine serum albumin. Then, cells were incubated with anti-collagen II antibody (Abcam) overnight at 4°C, Goat Anti-Mouse Alexa Fluor^®^ 647 secondary antibody (Abcam) was added for 1 h at 37°C in the dark and counterstained with DAPI. Finally, the photos were taken with fluorescence microscopy (OLYMPUS, IX51).

### Construction of CII-CAR

The gene encoding a scFv derived from a human anti-CII antibody (clone 551-3) was generated by splicing the variable region of the heavy chain to the variable region of the light chain *via* a (Gly_4_Ser)_3_ linker. This was cloned in-frame to the CD8*α* hinge and transmembrane domain. In this construct, the transmembrane domain is followed by a 4-1BB intracellular domain that serves as the co-stimulatory domain of the CAR, terminating with the CD3*ζ* intracellular activation domain. The complete CAR construct was sub-cloned into the lentiviral expression plasmid, pCDH-CMV-MCS-EF1α-copGFP (SBI) driven by a CMV promoter.

### Primary Human T Cell Lentivirus Transduction and CAR-T Cell Expansion

On day 0, 1.0 × 10^6^ T cells were cultured in 1.5 ml of X-VIVO 15 (Lonza) supplemented with 5% FBS (Gibco) and IL-2 (500 U/ml) and were stimulated with 100 ng/ml anti-human CD3 (clone OKT3) and anti-human CD28 (clone CD28.2). After 24 h, transduction was performed by first plating the lentivirus particle supernatants onto a 24-well culture plate pretreated with 20 μg/ml RetroNectin (Takara) and spinning at 2,000g and 32°C for 2 h, followed by centrifugation of the activated T cells onto the viral particle-coated plate at 1,000g and 32°C for 1 h. Virus medium was removed, and cells were re-suspended at 1 × 10^6^ cells/ml in fresh T cell medium. To determine the virus infection efficiency, CII-CAR was labeled with biotinylated protein L (Genscript) and PE Streptavidin (BD) on day 3 following transduction (30). Cells were allowed to expand in cultures until days 9 to 14. Cell cultures were monitored daily during expansion, and additional media were added to maintain a cell concentration of 1.0 × 10^6^ cells/ml. For all experiments using CII CAR-T cells, paired (from same donor) un-transduced T cells, activated and cultured for equivalent time, were served as control.

### Flow Cytometry

For phenotypic analysis we used CD8, CD4, and CD3 mAbs (BD) conjugated with FITC, PE or APC fluorochromes. Cell apoptosis was qualified using an annexin V-fluorescein isothiocyanate (FITC)/propidium iodide (PI) cell apoptosis kit (BD). Samples were analyzed with a BD FACS Calibur system equipped with the filter set for quadruple fluorescence signals and the Cell Quest software (BD). For each sample, we analyzed a minimum of 10,000 events.

### Analysis of Cytokine Production

The cytokine level detection was performed with a Cytometric Bead Array (CBA) Kit (BD). CII-CAR-T (2 × 10^5^ cells/well) were plated in the 96-well plates which were coated with collagen II (50 μg/ml), collagen I (50 μg/ml), C28/I2 cells, prepared fresh cartilage or FT-cartilage. After 24 h, supernatants from cell cultures were collected to test the cytokine level. In all experiments, T cells were also incubated in the 96-well plates coated with collagen II as control. The tests were performed according to the manufacturer’s protocols. The concentration of each cytokine was calculated based on standard curves generated from serial dilutions of cytokine standards provided by the manufacturer. Data were analyzed using the FCAP Array software (BD).

### Specific Lymphocyte Proliferation Test

CII-CAR-T or T cells (2 × 10^5^ cells/well) stained with CellTrace™ Far Red reagents were plated in the 96-well plates coated with collagen II (50 μg/ml) or two pieces of cartilage or C28/I2 cells without IL-2 for 3 days, CII-CAR-T or T cells alone were used as control. Cell proliferation was analyzed by flow cytometry analysis.

### Co-Culture of CII-CAR-T With C28/I2 Cells

C28/I2 cells were plated at a density of 20,000 cells per well in a 96-well plate and allowed to adhere overnight. 1 × 10^5^ CII-CAR-T cells were then added and allowed to incubate with C28/I2 cells. X-VIVO 15 supplemented with 5% FBS was added as control. After 24 h, supernatants from cell cultures were collected to test the cytokine level.

For apoptosis assays, C28/I2 cells were plated at a density of 4 × 10^5^ cells per well in a 6-well plate and allowed to adhere overnight. 2 × 10^6^ CII-CAR-T cells or T cells were then added and allowed to incubate with C28/I2 cells. X-VIVO 15 supplemented with 5% FBS was added as control. After 72 h, cells were collected and stained with CD3 before Annexin V-FITC and PI staining. T cells were excluded from the apoptosis analysis.

### Effects of Culture Supernatant of CII-CAR-T on Human Fresh Cartilage and C28/I2 Cells

CII-CAR-T (2 × 10^6^ cells/well) were plated in the 6-well plates which were coated with CII (50 μg/ml) for 24 h. Supernatants from cell cultures were centrifuged at 400 g for 5 min to pellet cells, collect the recovered supernatants in a syringe, and filter through a 0.45 μm filter (Millipore) to remove cellular debris. T cells’ culture supernatant was also used as control. For assessment of potential effects of the supernatants on human fresh cartilage and C28/I2 cells, they were treated with the supernatants for 24 h. For comparison, the FT-cartilage and 293T were used as control group. In addition, to confirm IL-6 can be produced by human fresh cartilage instead of FT-cartilage, supernatant was added in the cartilage group for 5 h, removed and washed with PBS three times, then replaced with the serum-free medium for 24 h to detect the level of IL-6 in the supernatant. To further explore the effects of TNF-α and IFN-*γ* on human fresh cartilage and C28/I2 cells, 5 ng/ml TNF-α or/and 10 ng/ml IFN-*γ* (sino biological) was added in culture medium for 24 h for detection.

### Statistics

All statistical analyses were performed using Prism software (v6.0, GraphPad Software, La Jolla, CA). Data are summarized as mean ± standard error mean (SEM). Student *t-*test was used to determine statistically significant differences between samples. When multiple comparison analyses were required, statistical significance was evaluated by one-way ANOVA. All *P*-values <0.05 were considered statistically significant.

## Results

### Characterization of CII-CAR Construct and Efficient Transduction of Primary Human T Cells

**Figure 1:**
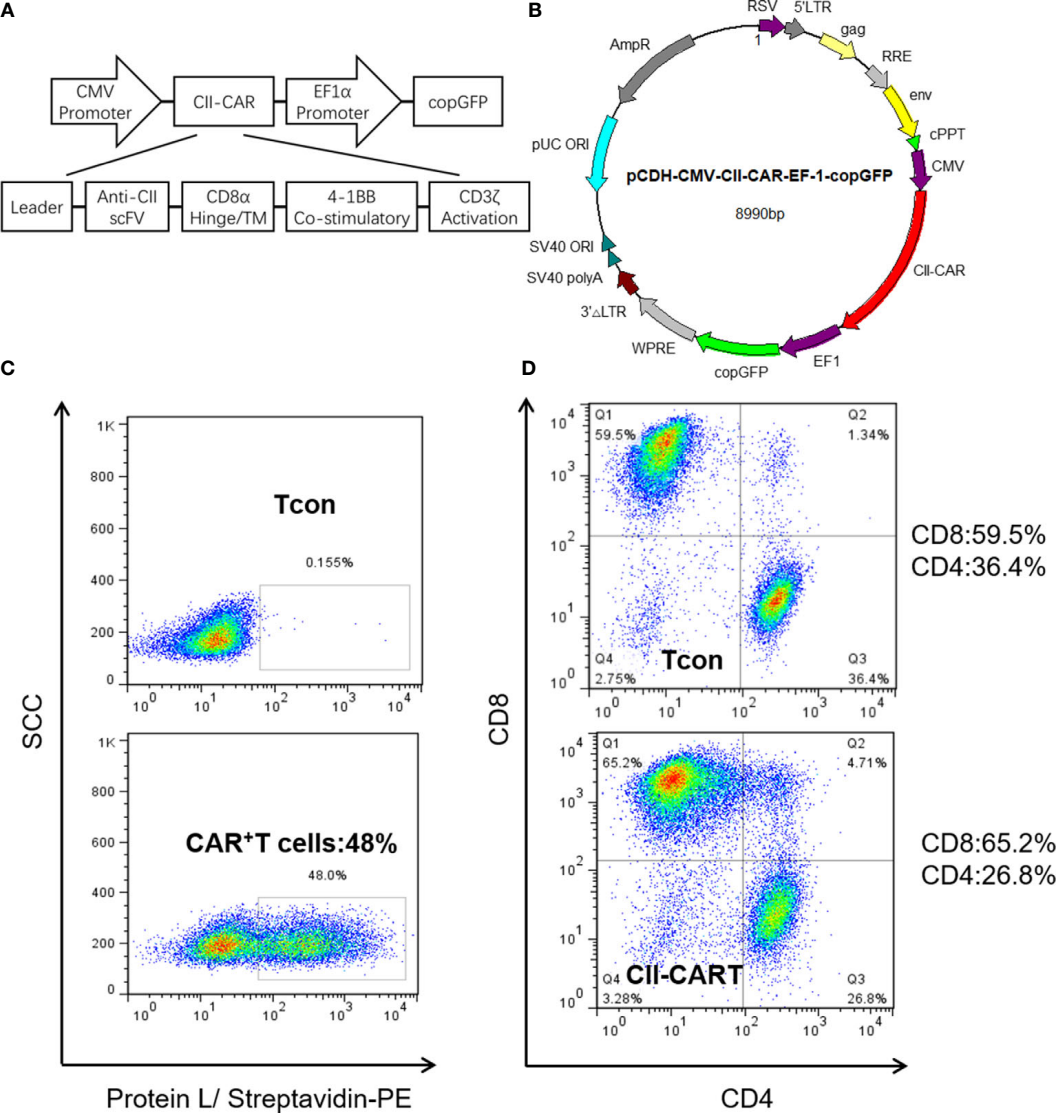
Construction and efficient transduction of CII-CAR. **(A)** CAR construct with CD8*α* leader, human anti-CII scFv, CD8*α* hinge and transmembrane domain, intracellular 4-1BB co-stimulatory domain, and intracellular CD3*ζ* activation domain. **(B)** The CAR construct was subcloned into a lentivirus expression plasmid following the CMV promoter and utilizing copGFP co-expression driven by the EF-1*α* promoter. **(C)** Efficient lentiviral transduction of primary human T cells encoding CII-CAR. **(D)** Similar CD4/CD8 ratios in control and CAR transduced T cells.

We generated a novel CII-CAR lentivirus vector incorporating a 4-1BB co-stimulatory domain and CD3*ζ* activation domain (**Figures 1A, B** and **Figure S1**). Primary human T cells were activated and then transduced with lentivirus encoding CII-CAR. Protein L is an immunoglobulin (Ig)-binding protein that binds to scFv and Fab fragments. Using protein L, CII-CAR transduced T cells exhibited specific staining pattern ranging from 20 to 50% of positive cells (**Figure 1C**). Similar CD4-to-CD8 ratios were observed between CII-CAR-transduced and control (un-transduced) T cells (**Figure 1D**). CII-CAR-T cells were expanded in cultures for 9–14 days.

### CII-CAR-T Cells Display Specific Cytokine Release and Proliferative Capacity When Stimulated by CII

**Figure 2:**
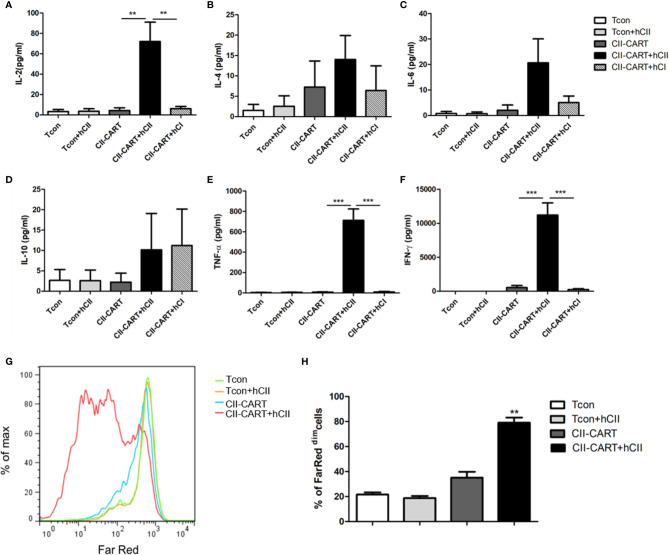
CII-CAR-T cells display specific cytokine release and proliferative capacity when stimulated by CII. **(A–F)** Supernatants were collected after 24 h co-culture of CII-CAR-T cells or Tcon in the presence of type I (50 μg/ml) or II collagen (50 μg/ml) and assayed for IL-2, IL-4, IL-6, IL-10, TNF-α, IFN-*γ* release by CBA. IL-2, TNF-α, and IFN-*γ* release were significantly increased compared with control group. Data are represented as mean ± SE of three independent experiments, *p < 0.05; **p < 0.01, ***p < 0.001. **(G, H)** CII-CAR-T or Tcon (1 × 10^5^ cells) were labeled with Far Red, then co-cultured with CII in the absence of CD3/28 mAbs and IL-2 for 3 days. The Far Red intensity in CII-CAR-T or Tcon cells was analyzed by flow cytometry, *p < 0.05; **p < 0.01, ***p < 0.001. Data are represented as mean ± SE of three independent experiments.

To determine whether the CII-CAR-T cells are specific for the proliferation and cytokine release to CII, we co-cultured conventional T cells (Tcon) or CII-CAR-T cells with type I or II collagen for 24h. Flow-based CBA assays demonstrated effector cytokine release of IL-2 (P < 0.01), IFN-*γ* (P < 0.001) and TNF-α (P < 0.001) were significantly increased when CII-CAR-T cells were stimulated with CII but not CI, whereas Tcon did not induce significant cytokine release (**Figures 2A–F**). Meanwhile, CII-CAR-T cells had also specific proliferative capacity to CII compared with CII-CAR-T cells alone (P < 0.01) (**Figures 2G, H**).

### CII-CAR-T Cells Display Specific Cytokine Release Capacity but No Obvious Effect on Inducing Apoptosis When Co-Cultured With Chondrocyte Cell Lines

**Figure 3:**
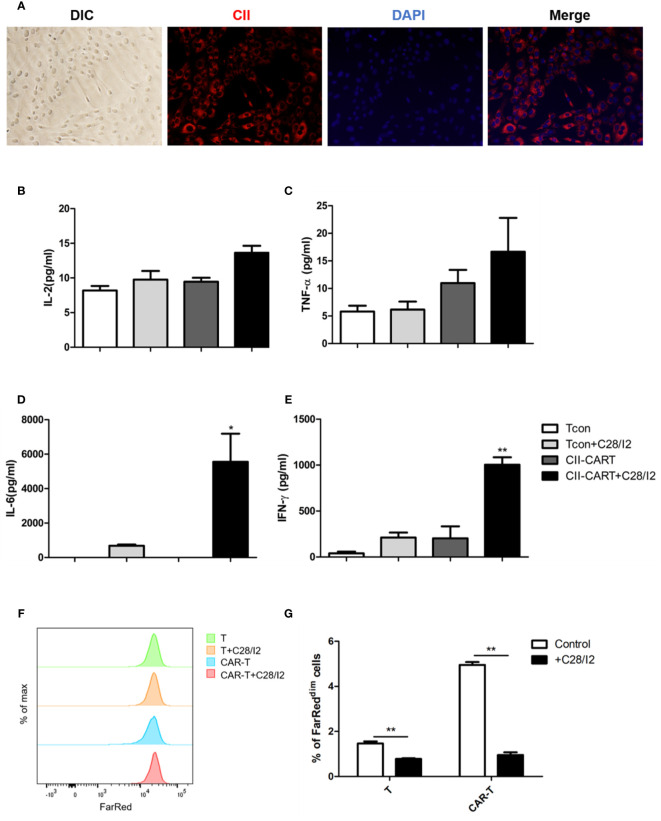
CII-CAR-T display specific cytokine release when co-cultured by C28/I2 cell line. **(A)** Immunofluorescence staining images show the expression of CII (red) and merged images (with DAPI, blue) in C28/I2. **(B–E)** Supernatants were collected after 24 h co-culture of CII-CAR-T cells or Tcon with C28/I2 and assayed for IL-2, IL-6, TNF-α, and IFN-*γ* release by CBA. **(F, G)** CII-CAR-T or Tcon cells (1 × 10^5^) were labeled with Far Red, then co-cultured with C28/I2 cells in the absence of CD3/28 mAbs and IL-2 for 3 days. CII-CAR-T or T cells alone were used as control. The Far Red intensity in CII-CAR-T or T cells was analyzed by flow cytometry. *p < 0.05; **p < 0.01. Data are represented as mean ± SE of three independent experiments.

Immunofluorescence staining images show that chondrocyte cell line C28/I2 expressed CII (**Figure 3A**). When CII-CAR-T cells were co-cultured with C28/I2 cells, the release of effector cytokines IL-6 and IFN-*γ* increased significantly, while no significant changes in IL-2 or TNF-α were observed (**Figures 3B–E**). To determine whether the CII-CAR-T cells can induce chondrocyte cells to apoptosis, we co-cultured T cells or CII-CAR-T cells at effector-to-target ratios (E:T = 5:1) with C28/I2 cells for 72 h. In flow-based assays experiment, no significant difference were detected in the apoptosis level of T cells or CII-CAR-T cells group compared with control group at 72 h (**Figure S2**). Additionally, proliferation of CII-CAR-T cells and T cells were inhibited when cocultured with C28/I2 cells *in vitro* (**Figures 3F, G**).

### CII-CAR-T Display Specific Cytokine Release and Proliferative Capacity When Stimulated by Human Fresh and FT-Cartilage

**Figure 4:**
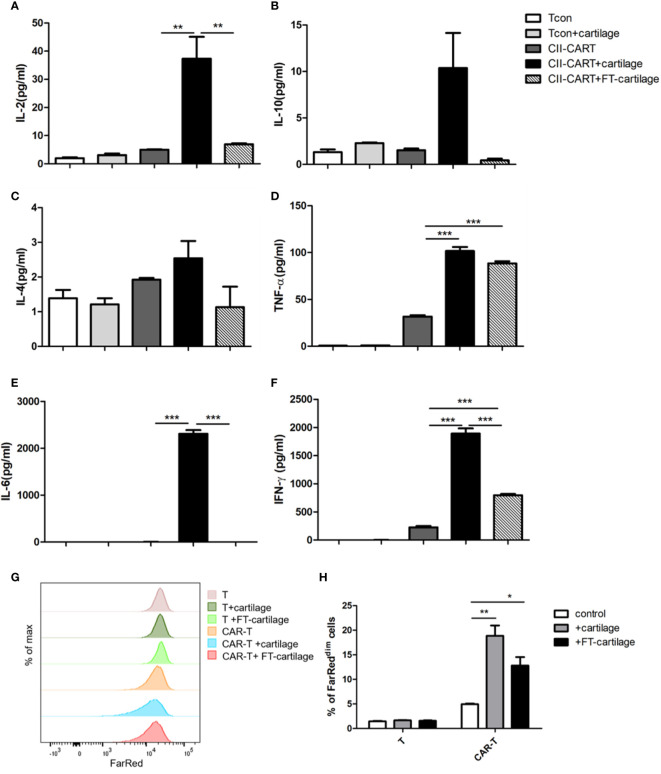
CII-CAR-T cells display specific cytokine release when stimulated by human fresh or FT-cartilage. **(A–F)**. Supernatants were collected after 24 h co-culture of CII-CAR-T cells or T con with fresh or FT-cartilage and assayed for IL-2, IL-4, IL-6, IL-10, TNF-α, and IFN-*γ* release by CBA. **(G–H)** CII-CAR-T or T cells (1 × 10^5^ cells) were labeled with Far Red, then co-cultured with fresh or FT-cartilage in the absence of CD3/28 mAbs and IL-2 for 4 days. CII-CAR-T or T cells alone were used as control. The Far Red intensity in CII-CAR-T cells or T cells was analyzed by flow cytometry. *p < 0.05; **p < 0.01; ***p < 0.001. Data are represented as mean ± SE of three independent experiments.

In order to determine whether CII-CAR-T cells will release inflammatory cytokines against human articular cartilage, circular pieces of fresh human articular cartilage were co-cultured with CII-CAR-T or Tcon for 24 h in culture medium containing 5% FBS without IL-2. It was found that concentrations of IL-2 (p < 0.01), IL-6 (p < 0.001), TNF-α (p < 0.001), and IFN-*γ* (p < 0.001) in supernatants of CII-CAR-T co-cultured with cartilage were significantly increased (**Figures 4A, D–F**). However, when FT-cartilage was co-cultured with CII-CAR-T cells for 24 h, it was found that levels of TNF-α (p < 0.001) and IFN-*γ* (p < 0.001) were still significantly higher, but level of IL-6 was almost not detected in the supernatant (**Figures 4D–F**). These results indicated that CII-CAR-T cells could release inflammatory cytokines against both fresh and FT-cartilage and indirectly indicated that living chondrocytes might participate in the release of IL-6, which might enhance the inflammation response. CII-CAR-T cells had also specific proliferative capacity to fresh (p < 0.01) or FT- cartilage (p < 0.05) (**Figures 4G, H**).

### Human Fresh Cartilage and C28/I2 Produce IL-6 When Stimulated by the Culture Supernatant of CII-CAR-T

**Figure 5:**
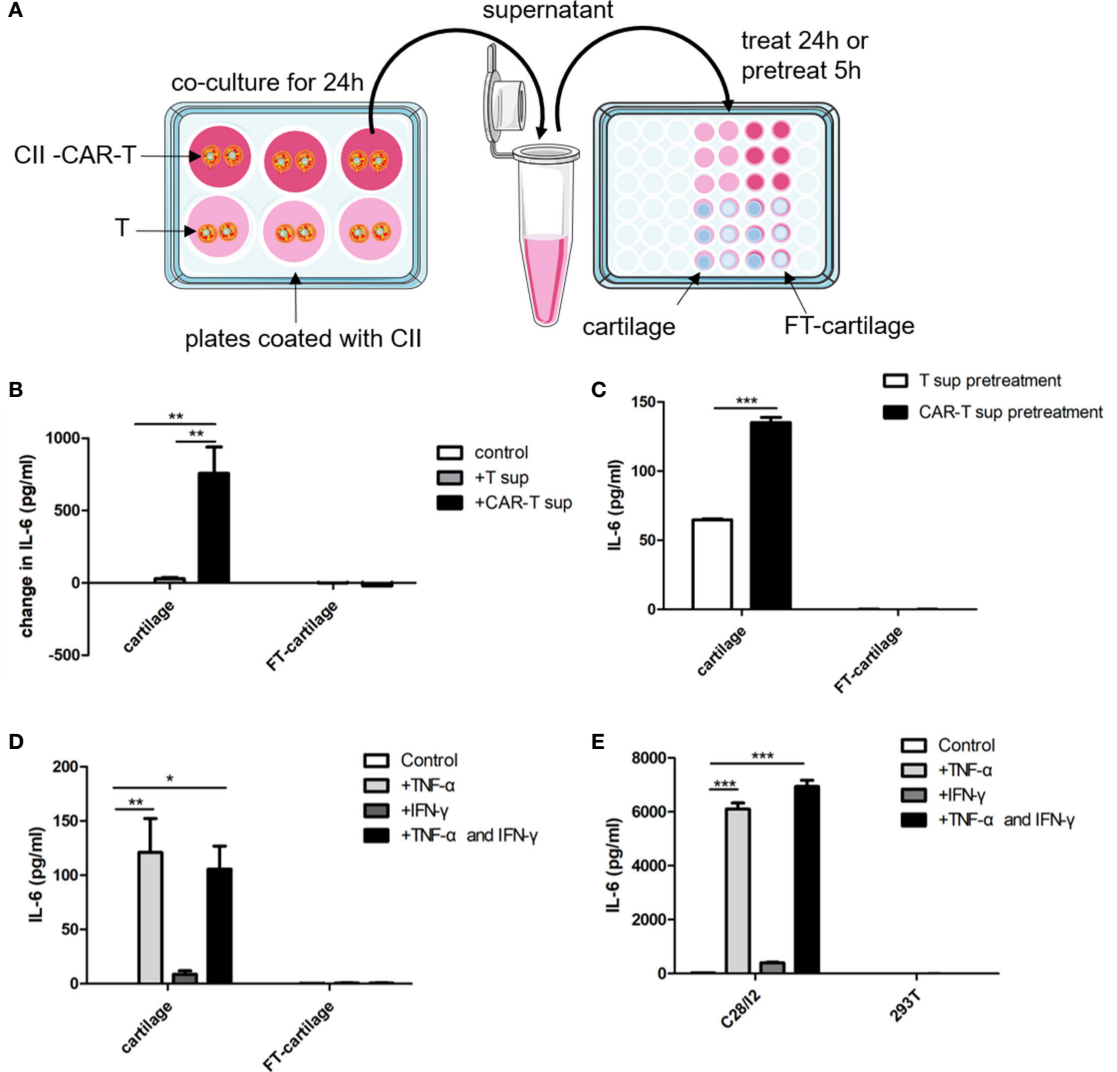
Human fresh cartilage and C28/I2 produce IL-6 when stimulated by CAR-T supernatants or cytokines. **(A)** Schematic representation of human fresh or FT-cartilage and C28/I2 were stimulated by CAR-T supernatants or cytokines. **(B)** Fresh or FT-cartilage was treated with supernatants of CII-CAR-T cells (CAR-T sup) or T cells (T sup) for 24 h, and IL-6 release was assayed by CBA. **(C)** IL-6 release in pretreatment groups was assayed by CBA. **(D)** IL-6 level produced by fresh or FT-cartilage when stimulated with 5 ng/ml TNF-α and/or 10 ng/ml IFN-*γ* for 24 h. **(E)** IL-6 level produced by C28/I2 and 293T cells when stimulated with 5 ng/ml TNF-α and/or 10 ng/ml IFN-*γ* for 24 h. Spontaneous release of cytokines by cartilage or C28/I2 was used as control. *p < 0.05; **p < 0.01; ***p < 0.001. Data are represented as mean ± SE of three independent experiments.

CII-CAR-T or T cells were co-cultured with CII in medium without IL-2 for 24 h. Human fresh or FT-cartilage samples were treated by supernatants from CII-CAR-T or T cell cultures for 24 h (**Figure 5A**). The results showed that the collected CAR-T supernatant can stimulate fresh cartilage to produce IL-6, and the level of IL-6 was significantly higher than that stimulated by T cell supernatant (**Figure 5B**) (p < 0.01). Furthermore, we used the supernatant to pretreat the cartilage samples for 5 h and replaced with serum-free medium to detect the level of IL-6 in the supernatant (**Figure 5A**). The results showed that IL-6 in pretreatment group with CII-CAR-T supernatant was significantly higher than the control group (**Figure 5C**) (p < 0.001). Finally, cartilage samples were directly stimulated with 5 ng/ml TNF-α and/or 10 ng/ml IFN-*γ* for 24 h. The results showed that TNF-α can stimulate fresh cartilage produce higher levels of IL-6 (p < 0.01) (**Figure 5D**), and the trends were similar in C28/I2 cells treated by TNF-α (p < 0.001) (**Figure 5E**). But the IL-6 level in the treatment group with TNF-α was significantly lower than the treatment group with culture supernatant of CII-activated CII-CAR-T (p < 0.05) (**Figure S3**). In addition, the IL-6 level in the FT-cartilage group was not increased significantly regardless of CII-CAR-T supernatant or TNF-α and/or 10 ng/ml IFN-*γ* treatment (**Figures 5B–E**).

## Discussion

Inflammation refers to a complex adaptive response by the host in response to tissue injury or xenobiotic insult. However, the physiological inflammatory response differs remarkably from the response that is typical of inflammatory arthritis (31). Inflammatory arthritis occurs in many diseases and is characterized by joint inflammation and damage (32, 33). An important point to note is that cartilage participates in both inflammatory and bone destruction phases (34). Chondrocytes are only resident cells in the cartilage, which not only act as target cells of inflammatory mediators, but also serve as effector cells and play an important role in cytokine cascades (15, 35–38). Insights into the cascades between chondrocytes and cartilage-reactive T cells could be helpful to find new biological markers and therapeutic targets of inflammatory arthritis. This study was aimed to develop an *in vitro* model that helps to explore the possible cytokine cascades between cartilage-reactive T cells and cartilage.

We generated universal cartilage-reactive T cells by CAR technology, and confirmed that CII-CAR-T cells display specific cytokine release including IL-2, IFN-*γ* and TNF-α and proliferative capacity when stimulated by CII but not CI. But when CII-CAR-T cells were co-cultured with C28/I2 cells, only IL-6 and IFN-*γ* was significantly increased, while there is no significant change in IL-2 or TNF-α level. Additionally, proliferation of CII-CAR-T cells and T cells was inhibited when cocultured with C28/I2 cells, which may be caused by inhibiting signal delivered when human articular chondrocyte upon contact with T cell (39). Thus, C28/I2 cells might not be a suitable component for the inflammatory model because CII-CAR-T cells were not appropriately activated against to C28/I2.

To solve this problem, we co-cultured CII-CAR-T with fresh cartilage, which is composed of extracellular matrix and only one cell type, the chondrocytes that synthesize the matrix (40). As expected, high levels of IL-2, IFN-*γ*, TNF-α, and IL-6 were detected in supernatant when CII-CAR-T co-cultured with fresh cartilage, but IL-6 was almost not detected in supernatant when CII-CAR-T co-cultured with FT-cartilage. CII-CAR-T cells also showed stronger proliferation ability in the presence of fresh or FT-cartilage. To further confirm that the chondrocytes in fresh cartilage respond to CII-CAR-T cells by secreting IL-6, culture supernatant of CII-activated CII-CAR-T which contains immune effector molecules was used to stimulate fresh cartilage, and changes in IL-6 levels was significantly increased compared with control groups.

Finally, when cartilage samples were stimulated directly with TNF-α and/or IFN-*γ*, it showed TNF-α could stimulate chondrocytes to produce IL-6, but synergy is not present between TNF-α and IFN-*γ*. However, the IL-6 level in the treatment group with TNF-α was significantly lower than the treatment group with culture supernatant of CII-activated CII-CAR-T, which indicated that CII-CAR-T could produce other effector molecules to stimulate chondrocytes to produce stronger inflammatory responses. At least, IL-2, IL-6, TNF-α, IFN-*γ* were produced when co-cultures of CII-CAR-T cells and fresh cartilage, and these cytokines also play roles in the occurrence and development of inflammatory arthritis and other autoimmune diseases (19, 20).

**Figure 6:**
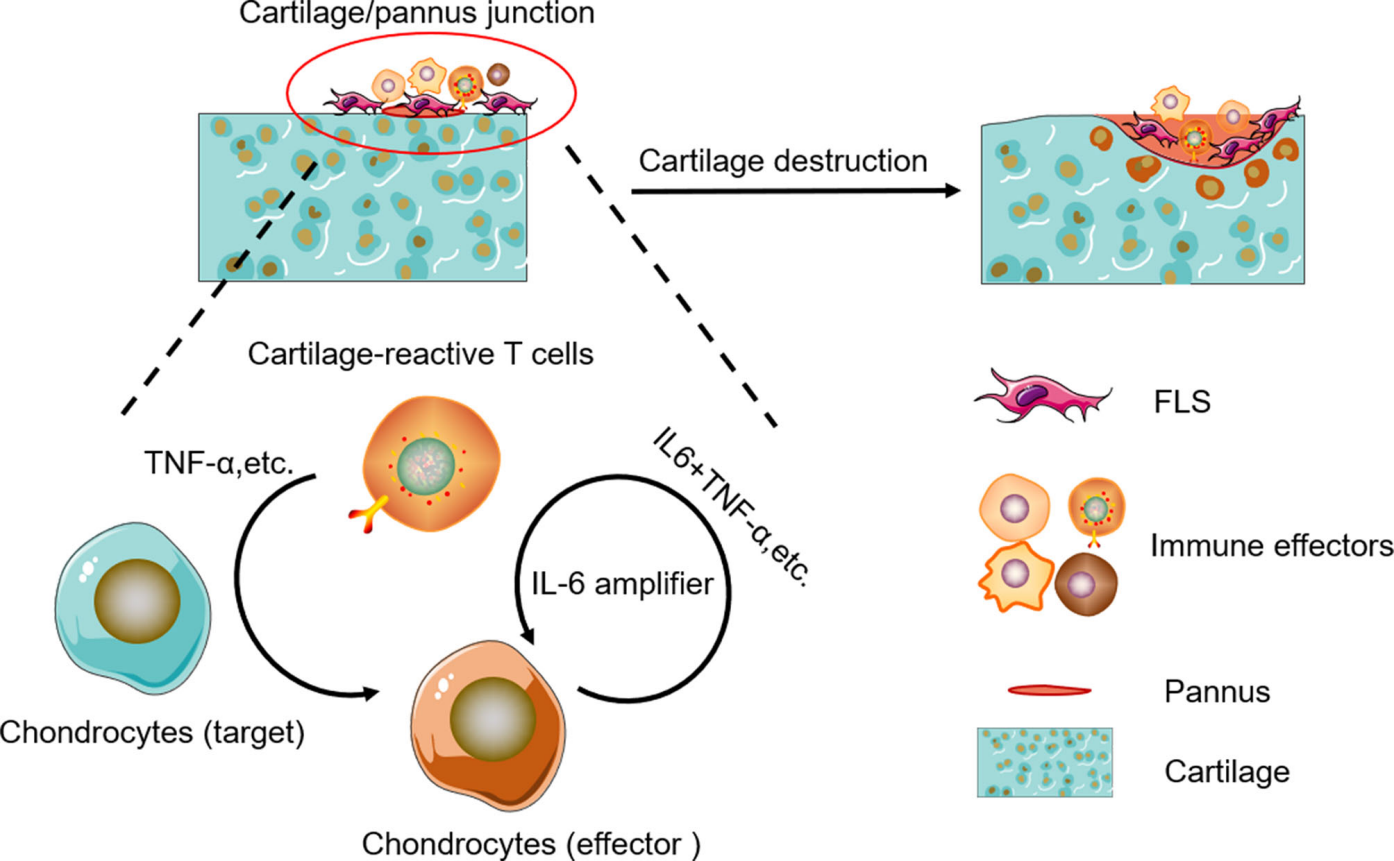
Schematic representation of cytokine cascade and the positive feedback loop between autoreactive T cells and chondrocytes.

In RA, TNF-α and IL-6 are the two well-known cytokines triggering synovitis and bone erosions. TNF-α is clearly a central cytokine in RA pathophysiology, which has a pivotal role in the initiation and amplification of the cytokine cascade (41–45), and mediate a wide variety of effector functions relevant to the pathogenesis of RA, including leukocyte and endothelial activation, synoviocyte activation and survival, cytokine and chemokine amplification, angiogenesis, and nociceptor activation (46, 47). IL-6 is also a key cytokine in RA pathogenesis and mediates pleiotropic functions rather similar to those of TNF-α in the synovial environment (46, 47). Unlike a number of other cytokines, IL-6 can activate cells through both membrane-bound (IL-6R) and soluble receptors (sIL-6R), thus widening the number of cell types responsive to this cytokine (48–51). IL-6-mediated inflammation amplifier was reported as a key molecular mechanism in chronic inflammation (15, 52, 53), which triggers a vicious circle of escalating RA disease activity (51, 54, 55). IL-6 plus TNF-α or IL-6 plus IL-17 can trigger IL-6 amplifier, leading to positive feedback for IL-6 signaling (15). In this model, CII-CAR-T produced TNF-α against cartilage samples, then TNF-α could induce chondrocyte produce IL-6, finally IL-6 plus TNF-α can lead to activation of IL-6 amplifier (**Figure 6**). However, there are still other effectors in the supernatant of CII-CAR-T that participate in the inflammation response, and the model may serve as a useful tool to research unknown cytokine cascades and synergy.

As *in vitro* models can only be used to investigate limited numbers of cell type, it would be much better to explore the immune cascades *in vivo* by using murine CII-CAR-T cells to target murine cartilage protein. Although it might not be the best to mimic the complex cellular and molecular interactions involved in T lymphocyte response in arthritis, this *in vitro* model is rapid, simple, and intuitive, which can be used as a suitable tool to for studying the cytokine cascades caused by the interaction between cartilage-reactive T cells and chondrocytes. The study of CAR-T mediated cytokine responses in chondrocytes may also contribute to the understanding of serious arthritis symptoms caused by CAR-T therapy, and more attention should be paid when CAR-T is used to treat bone metastases or bone cancer. In addition, while CII-CAR to be expressed on effector T cells can serve as a tool to study inflammation, the expression on regulator T cells might be used as a novel treatment for inflammatory arthritis (56, 57).

## Conclusion

In this study, the rapid inflammatory model was established, and it could intuitively reflect the cytokine cascade of TNF-α and IL-6. However, we only examined limited variety of cytokines, and more detailed detection should be conducted in the future. This inflammatory model will be helpful in discovering other cytokine cascades, which should contribute to find new markers and therapeutic targets of inflammatory arthritis.

## Data Availability Statement

The original contributions presented in the study are included in the article/**Supplementary Material**. Further inquiries can be directed to the corresponding authors.

## Ethics Statement

The studies involving human participants were reviewed and approved by The Ethics Committee of First Affiliated Hospital of Harbin Medical University. The patients/participants provided their written informed consent to participate in this study.

## Author Contributions

Writing—original draft preparation: XL and JuZ. Methodology: CS, ZL, YL, DY. Resources, JD, HS, and LK. Data curation: JW. Funding acquisition: FC, JiZ, and ZW. Writing—review and editing: ZW and SZ. Project administration, ZW and SZ. All authors contributed to the article and approved the submitted version.

## Conflict of Interest

The authors declare that the research was conducted in the absence of any commercial or financial relationships that could be construed as a potential conflict of interest.

## References

[1] Smilek DE Ehlers MR Nepom GT Restoring the balance: immunotherapeutic combinations for autoimmune disease. Dis Model Mech (2014) 7(5):503–13. 10.1242/dmm.015099 PMC4007402 24795433

[2] Firestein GS McInnes IB Immunopathogenesis of Rheumatoid Arthritis. Immunity (2017) 46(2):183–96. 10.1016/j.immuni.2017.02.006 PMC5385708 28228278

[3] Zou Y Xu S Xiao Y Qiu Q Shi M Wang J Long noncoding RNA LERFS negatively regulates rheumatoid synovial aggression and proliferation. J Clin Invest (2018) 128(10):4510–24. 10.1172/JCI97965 PMC6159954 30198906

[4] Yap HY Tee SZ Wong MM Chow SK Peh SC Teow SY Pathogenic Role of Immune Cells in Rheumatoid Arthritis: Implications in Clinical Treatment and Biomarker Development. Cells (2018) 7(10):161. 10.3390/cells7100161 PMC6211121 30304822

[5] Bi X Guo XH Mo BY Wang ML Luo XQ Chen YX LncRNA PICSAR promotes cell proliferation, migration and invasion of fibroblast-like synoviocytes by sponging miRNA-4701-5p in rheumatoid arthritis. EBioMedicine (2019) 50:408–20. 10.1016/j.ebiom.2019.11.024 PMC6921299 31791845

[6] Kong N Lan Q Su W Chen M Wang J Yang Z Induced T regulatory cells suppress osteoclastogenesis and bone erosion in collagen-induced arthritis better than natural T regulatory cells. Ann Rheum Dis (2012) 71(9):1567–72. 10.1136/annrheumdis-2011-201052 PMC4038329 22764040

[7] Cope AP T cells in rheumatoid arthritis. Arthritis Res Ther (2008) 10(Suppl 1):S1. 10.1186/ar2412 PMC2582813 19007421

[8] Auger I Balandraud N Massy E Hemon M Peen E Arnoux F Peptidyl arginine deiminase autoimmunity and the development of ACPA in rheumatoid arthritis. The “hapten carrier” model. Arthritis Rheumatol (2019) 72(6):903–11. 10.1002/art.41189 PMC7317357 31820586

[9] Musters A Klarenbeek PL Doorenspleet ME Balzaretti G Esveldt REE van Schaik BDC In Rheumatoid Arthritis, Synovitis at Different Inflammatory Sites Is Dominated by Shared but Patient-Specific T Cell Clones. J Immunol (2018) 201(2):417–22. 10.4049/jimmunol.1800421 29891556

[10] Yang M Liu Y Mo B Xue Y Ye C Jiang Y Helios but not CD226, TIGIT and Foxp3 is a Potential Marker for CD4(+) Treg Cells in Patients with Rheumatoid Arthritis. Cell Physiol Biochem (2019) 52(5):1178–92. 10.33594/000000080 PMC6943339 30990587

[11] Hu XX Wu YJ Zhang J Wei W T-cells interact with B cells, dendritic cells, and fibroblast-like synoviocytes as hub-like key cells in rheumatoid arthritis. Int Immunopharmacol (2019) 70:428–34. 10.1016/j.intimp.2019.03.008 30856393

[12] Xu A Liu Y Chen W Wang J Xue Y Huang F TGF-beta-Induced Regulatory T Cells Directly Suppress B Cell Responses through a Noncytotoxic Mechanism. J Immunol (2016) 196(9):3631–41. 10.4049/jimmunol.1501740 PMC4868785 27001954

[13] Chen M Lin X Liu Y Li Q Deng Y Liu Z The function of BAFF on T helper cells in autoimmunity. Cytokine Growth Factor Rev (2014) 25(3):301–5. 10.1016/j.cytogfr.2013.12.011 PMC4055514 24411564

[14] Ramalingam R Larmonier CB Thurston RD Midura-Kiela MT Zheng SG Ghishan FK Dendritic cell-specific disruption of TGF-beta receptor II leads to altered regulatory T cell phenotype and spontaneous multiorgan autoimmunity. J Immunol (2012) 189(8):3878–93. 10.4049/jimmunol.1201029 PMC3466393 22972928

[15] Ota M Tanaka Y Nakagawa I Jiang JJ Arima Y Kamimura D Role of Chondrocytes in the Development of Rheumatoid Arthritis Via Transmembrane Protein 147-Mediated NF-kappaB Activation. Arthritis Rheumatol (2020) 72(6):931–94. 10.1002/art.41182 31785076

[16] June CH O'Connor RS Kawalekar OU Ghassemi S Milone MC CAR T cell immunotherapy for human cancer. Science (2018) 359(6382):1361–5. 10.1126/science.aar6711 29567707

[17] Roybal KT Refining cell therapy. Science (2018) 359(6380):1112–3. 10.1126/science.aat0962 29590036

[18] Hay KA Cytokine release syndrome and neurotoxicity after CD19 chimeric antigen receptor-modified (CAR-) T cell therapy. Br J Haematol (2018) 183(3):364–74. 10.1111/bjh.15644 30407609

[19] Saklatvala J Tumour necrosis factor alpha stimulates resorption and inhibits synthesis of proteoglycan in cartilage. Nature (1986) 322(6079):547–9. 10.1038/322547a0 PMC7095107 3736671

[20] Yang S Xie C Chen Y Wang J Chen X Lu Z Differential roles of TNFalpha-TNFR1 and TNFalpha-TNFR2 in the differentiation and function of CD4(+)Foxp3(+) induced Treg cells in vitro and in vivo periphery in autoimmune diseases. Cell Death Dis (2019) 10(1):27. 10.1038/s41419-018-1266-6 30631042 PMC6328545

[21] Wang LX Chen X Jia M Wang S Shen J Arthritis of large joints shown as a rare clinical feature of cytokine release syndrome after chimeric antigen receptor T cell therapy: A case report. Medicine (Baltimore) (2018) 97(16):e0455. 10.1097/MD.0000000000010455 29668614 PMC5916644

[22] Nandakumar KS Targeting IgG in Arthritis: Disease Pathways and Therapeutic Avenues. Int J Mol Sci (2018) 19(3):677. 10.3390/ijms19030677 PMC5877538 29495570

[23] Cook AD Rowley MJ Mackay IR Gough A Emery P Antibodies to type II collagen in early rheumatoid arthritis. Correlation with disease progression. Arthritis Rheum (1996) 39(10):1720–7. 10.1002/art.1780391015 8843863

[24] Zhou L Wang J Li J Li T Chen Y June RR 1,25-Dihydroxyvitamin D3 Ameliorates Collagen-Induced Arthritis via Suppression of Th17 Cells Through miR-124 Mediated Inhibition of IL-6 Signaling. Front Immunol (2019) 10:178. 10.3389/fimmu.2019.00178 30792721 PMC6374300

[25] Brennan FM McInnes IB Evidence that cytokines play a role in rheumatoid arthritis. J Clin Invest (2008) 118(11):3537–45. 10.1172/JCI36389 PMC2575731 18982160

[26] Zhou S Thornhill TS Meng F Xie L Wright J Glowacki J Influence of osteoarthritis grade on molecular signature of human cartilage. J Orthop Res (2016) 34(3):454–62. 10.1002/jor.23043 26336057

[27] Outerbridge RE The etiology of chondromalacia patellae. J Bone Joint Surg Br (1961) 43-B:752–7. 10.1302/0301-620X.43B4.752 14038135

[28] Clements KM Bee ZC Crossingham GV Adams MA Sharif M How severe must repetitive loading be to kill chondrocytes in articular cartilage? Osteoarthritis Cartilage (2001) 9(5):499–507. 10.1053/joca.2000.0417 11467899

[29] Zhou C Zheng H Buckwalter JA Martin JA Enhanced phagocytic capacity endows chondrogenic progenitor cells with a novel scavenger function within injured cartilage. Osteoarthritis Cartilage (2016) 24(9):1648–55. 10.1016/j.joca.2016.04.016 PMC4992612 27130155

[30] Zheng Z Chinnasamy N Morgan RA Protein L: a novel reagent for the detection of chimeric antigen receptor (CAR) expression by flow cytometry. J Transl Med (2012) 10:29. 10.1186/1479-5876-10-29 22330761 PMC3299624

[31] Perretti M Cooper D Dalli J Norling LV Immune resolution mechanisms in inflammatory arthritis. Nat Rev Rheumatol (2017) 13(2):87–99. 10.1038/nrrheum.2016.193 28053331

[32] Komatsu N Takayanagi H Inflammation and bone destruction in arthritis: synergistic activity of immune and mesenchymal cells in joints. Front Immunol (2012) 3:77. 10.3389/fimmu.2012.00077 22566958 PMC3342288

[33] Busch R Kollnberger S Mellins ED HLA associations in inflammatory arthritis: emerging mechanisms and clinical implications. Nat Rev Rheumatol (2019) 15(6):364–81. 10.1038/s41584-019-0219-5 31092910

[34] Li N Wang JC Liang TH Zhu MH Wang JY Fu XL Pathologic finding of increased expression of interleukin-17 in the synovial tissue of rheumatoid arthritis patients. Int J Clin Exp Pathol (2013) 6(7):1375–9. PMC3693203 23826419

[35] Otero M Goldring MB Cells of the synovium in rheumatoid arthritis. Chondrocytes. Arthritis Res Ther (2007) 9(5):220. 10.1186/ar2292 18001488 PMC2212563

[36] Koskinen A Vuolteenaho K Nieminen R Moilanen T Moilanen E Leptin enhances MMP-1, MMP-3 and MMP-13 production in human osteoarthritic cartilage and correlates with MMP-1 and MMP-3 in synovial fluid from OA patients. Clin Exp Rheumatol (2011) 29(1):57–64. 10.1016/S1063-4584(10)60038-6 21345293

[37] Conde J Otero M Scotece M Abella V Gómez R López V E74-Like Factor (ELF3) and Leptin, a Novel Loop Between Obesity and Inflammation Perpetuating a Pro-Catabolic State in Cartilage. Cell Physiol Biochem (2018) 45(6):2401–10. 10.1159/000488227 29550824

[38] Tseng CC Chen YJ Chang WA Tsai WC Ou TT Wu CC Dual Role of Chondrocytes in Rheumatoid Arthritis: The Chicken and the Egg. Int J Mol Sci (2020) 21(3):1071. 10.3390/ijms21031071 PMC7038065 32041125

[39] Pereira RC Martinelli D Cancedda R Gentili C Poggi A Human Articular Chondrocytes Regulate Immune Response by Affecting Directly T Cell Proliferation and Indirectly Inhibiting Monocyte Differentiation to Professional Antigen-Presenting Cells. Front Immunol (2016) 7:415. 10.3389/fimmu.2016.00415 27822208 PMC5075572

[40] Sophia Fox AJ Bedi A Rodeo SA The basic science of articular cartilage: structure, composition, and function. Sports Health (2009) 1(6):461–8. 10.1177/1941738109350438 PMC3445147 23015907

[41] Butler DM Maini RN Feldmann M Brennan FM Modulation of proinflammatory cytokine release in rheumatoid synovial membrane cell cultures. Comparison of monoclonal anti TNF-alpha antibody with the interleukin-1 receptor antagonist. Eur Cytokine Netw (1995) 6(4):225–30. 8789287

[42] Alvaro-Gracia JM Zvaifler NJ Brown CB Kaushansky K Firestein GS Cytokines in chronic inflammatory arthritis. VI. Analysis of the synovial cells involved in granulocyte-macrophage colony-stimulating factor production and gene expression in rheumatoid arthritis and its regulation by IL-1 and tumor necrosis factor-alpha. J Immunol (1991) 146(10):3365–71. 2026869

[43] Haworth C Brennan FM Chantry D Turner M Maini RN Feldmann M Expression of granulocyte-macrophage colony-stimulating factor in rheumatoid arthritis: regulation by tumor necrosis factor-alpha. Eur J Immunol (1991) 21(10):2575–9. 10.1002/eji.1830211039 1915559

[44] Feldmann M Development of anti-TNF therapy for rheumatoid arthritis. Nat Rev Immunol (2002) 2(5):364–71. 10.1038/nri802 12033742

[45] Terrando N Monaco C Ma D Foxwell BM Feldmann M Maze M Tumor necrosis factor-alpha triggers a cytokine cascade yielding postoperative cognitive decline. Proc Natl Acad Sci USA (2010) 107(47):20518–22. 10.1073/pnas.1014557107 PMC2996666 21041647

[46] Fang Q Sun YY Cai W Dodge GR Lotke PA Williams WV Cartilage-reactive T cells in rheumatoid synovium. Int Immunol (2000) 12(5):659–69. 10.1093/intimm/12.5.659 10784612

[47] Noack M Miossec P Selected cytokine pathways in rheumatoid arthritis. Semin Immunopathol (2017) 39(4):365–83. 10.1007/s00281-017-0619-z 28213794

[48] Dayer JM Choy E Therapeutic targets in rheumatoid arthritis: the interleukin-6 receptor. Rheumatol (Oxford) (2010) 49(1):15–24. 10.1093/rheumatology/kep329 PMC2789585 19854855

[49] Zheng SG Wang J Horwitz DA Cutting edge: Foxp3+CD4+CD25+ regulatory T cells induced by IL-2 and TGF-beta are resistant to Th17 conversion by IL-6. J Immunol (2008) 180(11):7112–6. 10.4049/jimmunol.180.11.7112 18490709

[50] Lu L Lan Q Li Z Zhou X Gu J Li Q Critical role of all-trans retinoic acid in stabilizing human natural regulatory T cells under inflammatory conditions. Proc Natl Acad Sci U S A (2014) 111(33):E3432–40. 10.1073/pnas.1408780111 PMC4143025 25099355

[51] Luo Y Zheng SG Hall of Fame among Pro-inflammatory Cytokines: Interleukin-6 Gene and Its Transcriptional Regulation Mechanisms. Front Immunol (2016) 7:604. 10.3389/fimmu.2016.00604 28066415 PMC5165036

[52] Murakami M Hirano T The pathological and physiological roles of IL-6 amplifier activation. Int J Biol Sci (2012) 8(9):1267–80. 10.7150/ijbs.4828 PMC3491450 23136555

[53] Murakami M Hirano T A four-step model for the IL-6 amplifier, a regulator of chronic inflammations in tissue-specific MHC class II-associated autoimmune diseases. Front Immunol (2011) 2:22. 10.3389/fimmu.2011.00022 22566812 PMC3341963

[54] Schinnerling K Aguillón JC Catalán D Soto L The role of interleukin-6 signalling and its therapeutic blockage in skewing the T cell balance in rheumatoid arthritis. Clin Exp Immunol (2017) 189(1):12–20. 10.1111/cei.12966 28369786 PMC5461092

[55] Ogata A Kato Y Higa S Yoshizaki K IL-6 inhibitor for the treatment of rheumatoid arthritis: A comprehensive review. Mod Rheumatol (2019) 29(2):258–67. 10.1080/14397595.2018.1546357 30427250

[56] Zhang Q Lu W Liang CL Chen Y Liu H Qiu F Chimeric Antigen Receptor (CAR) Treg: A Promising Approach to Inducing Immunological Tolerance. Front Immunol (2018) 9:2359. 10.3389/fimmu.2018.02359 30369931 PMC6194362

[57] Wang Z Liu X Cao F Bellanti JA Zhou J Zheng SG Prospects of the Use of Cell Therapy to Induce Immune Tolerance. Front Immunol (2020) 11:792. 10.3389/fimmu.2020.00792 32477335 PMC7235417

