# Supplementary material for: Construction of CII-Specific CAR-T to Explore the Cytokine Cascades Between Cartilage-Reactive T Cells and Chondrocytes
Source: Front Immunol. 2020 Dec 4;11:568741. doi: 10.3389/fimmu.2020.568741 (PMC7746615; doi:10.3389/fimmu.2020.568741)
Supplement: Supplementary file 1 [file DataSheet_1.docx]

**Supplementary Material**

**
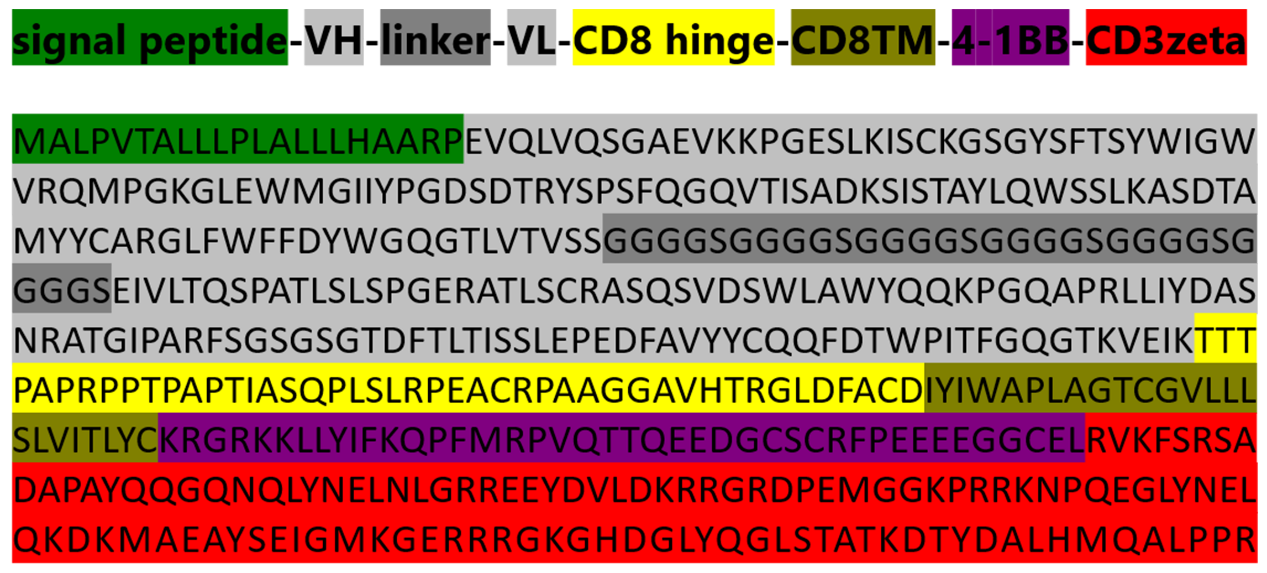
**

**Figure S1 Amino acid sequence of CII-CAR.**


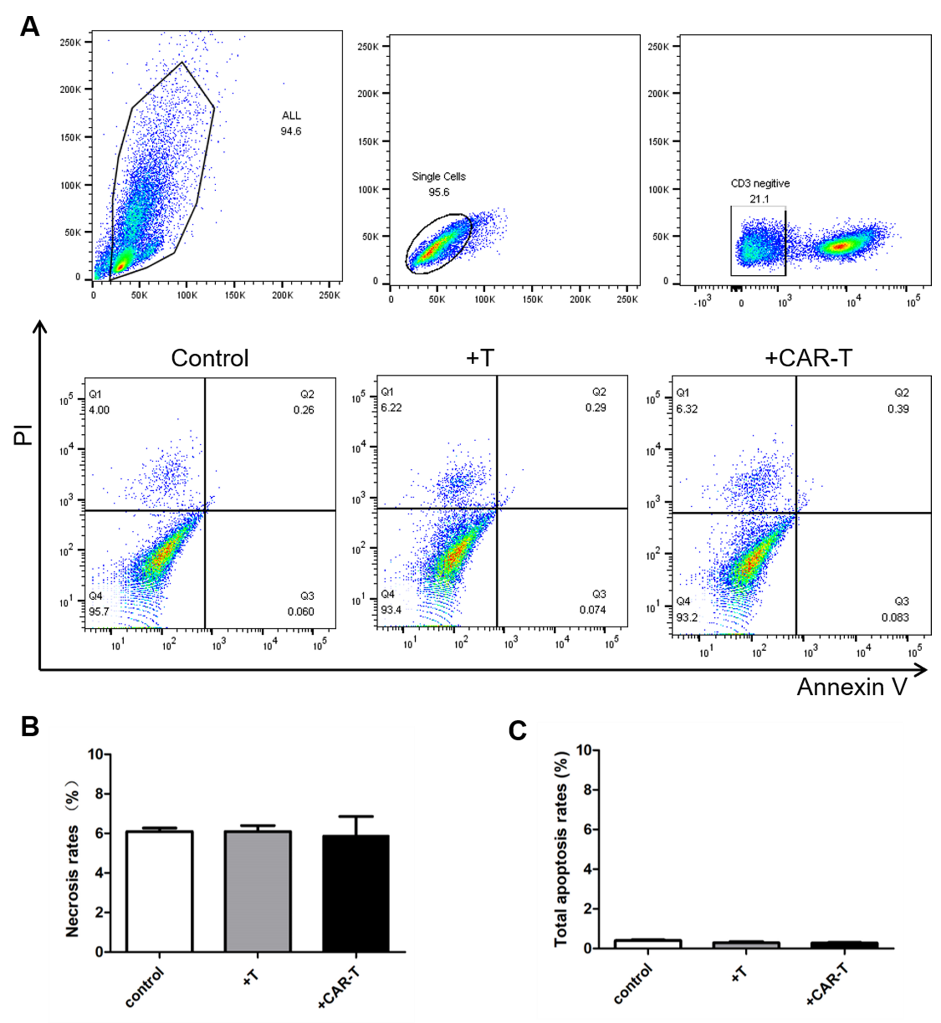


**Figure S2 Effect of CII-CAR-T Cells on Inducing Apoptosis When Co-Cultured with C28/I2**

(A) Representative flow cytometry data to illustrate gating and quadrant strategy for FACS measurement of apoptosis. No obvious effect on inducing (B) apoptosis or (C) necrosis was when co-cultured with C28/I2. Data are represented as mean ± SE of 3 independent experiments.


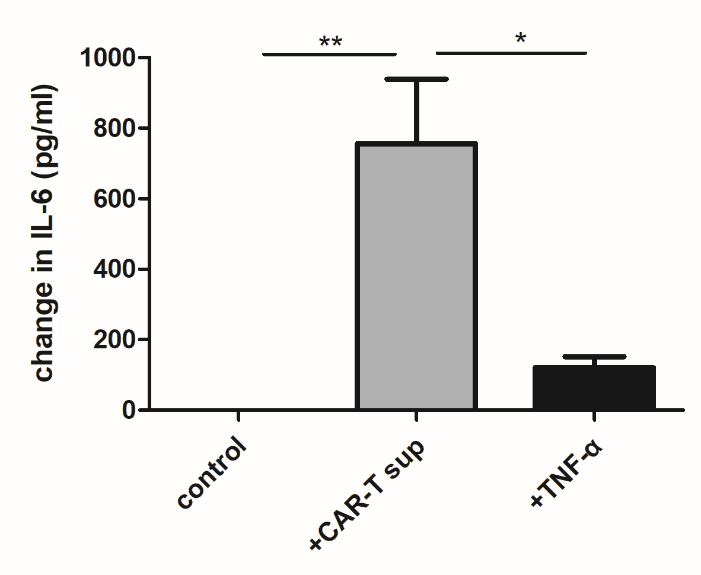


**Figure S3 Changes in IL-6 levels were compared between the treatment groups.**

Changes in IL-6 levels in the treatment group with TNF-α or culture supernatant of CII-activated CII-CAR-T, spontaneous release of IL-6 by cartilage were used as control. *p < 0.05; **p < 0.01. Data are represented as mean ± SE of 3 independent experiments.
